# Variable but Inheritable: Colchicine-Induced Polyploidy in *Phaseolus vulgaris* Generates Physiological Variability with Limited Inheritance

**DOI:** 10.3390/plants15162464

**Published:** 2026-08-14

**Authors:** Vera Martínez-Barradas, Gabriela Olivo-Vidal, Rodrigo Mora-Sanhueza, Francisco Barco-Rubio, Gabriela Jarpa-Tauler, René Morales, Marjorie Reyes-Díaz, Ricardo Tighe-Neira, Claudio Inostroza-Blancheteau, Patricio Arce-Johnson

**Affiliations:** 1 Instituto de Ciencias Aplicadas, Facultad de Ingeniería, Universidad Autónoma de Chile, Santiago 8580640, Chile; vera.martinez.b@uautonoma.cl (V.M.-B.); gfolivo@alumni.uc.cl (G.O.-V.); fbarco@uc.cl (F.B.-R.); remorales1@uc.cl (R.M.); 2 Programa de Doctorado en Ciencias Agropecuarias, Facultad de Recursos Naturales, Universidad Católica de Temuco, Temuco 4781312, Chile; rmora2023@alu.uct.cl (R.M.-S.); claudio.inostroza@uct.cl (C.I.-B.); 3 Laboratorio de Fisiología y Biotecnología Vegetal, Departamento de Ciencias Agropecuarias y Acuícolas, Facultad de Recursos Naturales, Universidad Católica de Temuco, Temuco, 4781312, Chile; rtighe@uct.cl; 4 Facultad de Agronomía y Sistemas Naturales, Pontificia Universidad Católica de Chile, Santiago 6904411, Chile; 5 Facultad de Ciencias Biológicas, Pontificia Universidad Católica de Chile, Santiago 8320000, Chile; gjarpa@uc.cl; 6 Laboratorio de Ecofisiología Molecular y Funcional de Plantas, Departamento de Ciencias Químicas y Recursos Naturales, Facultad de Ingeniería y Ciencias, Universidad de La Frontera, Temuco 4811230, Chile; marjorie.reyes@ufrontera.cl; 7 Nucleo de Investigación en Producción Alimentaria, Facultad de Recursos Naturales, Universidad Católica de Temuco, Temuco 4781312, Chile; 8 Agencia Nacional de Investigación y Desarrollo, Millennium Nucleus in Data Science for Plant Resilience (Phytolearning), Santiago 8370186, Chile

**Keywords:** *Phaseolus vulgaris*, colchicine, induced polyploidy, tetraploids

## Abstract

Polyploidy is widely used in plant breeding to increase phenotypic variation and potentially improve stress-related traits; however, its effects in common bean remain poorly characterized. Here, colchicine-induced polyploidy was evaluated in *Phaseolus vulgaris* cv. Zorzal to determine whether tetraploid lines exhibit coordinated changes in stomatal anatomy, physiology, pigment composition, oxidative balance, and ploidy inheritance. Polyploidy was induced using different colchicine exposure regimes and confirmed by flow cytometry. Stomatal traits, gas-exchange parameters, photosynthetic pigments, oxidative stress markers, antioxidant capacity, and multivariate phenotypic relationships were subsequently analyzed in confirmed tetraploid lines. Colchicine treatments successfully generated tetraploid and mixoploid plants, although induction efficiency was highly variable among replicates. Tetraploid lines showed heterogeneous physiological responses, including increases in the photosynthetic rate and stomatal conductance in some genotypes, but no consistent reduction in stomatal pore area index. Pigment composition and antioxidant-related traits also varied among lines, without a uniform response pattern. Multivariate analyses revealed coordinated but highly divergent phenotypic profiles among tetraploid genotypes. Importantly, the induced tetraploids were not stably transmitted to the C1 generation, with a predominance of diploid progeny among the evaluated offspring. Overall, colchicine-induced polyploidization generated substantial physiological variability among independently derived tetraploid plants of *P. vulgaris* cv. ‘Zorzal’; however, the predominance of diploid individuals among the evaluated C1 progeny indicates limited transmission of the induced tetraploid state to the subsequent generation.

## 1. Introduction

Polyploidy refers to the condition in which organisms have more than two complete sets of chromosomes within their cell nucleus, arising from whole-genome duplication events caused by failures in chromosome segregation and subsequent cytokinesis during cell division [1,2,3].

Polyploidy is a common genetic process among plants. It may occur naturally or be artificially induced. Throughout evolution, multiple plant lineages have undergone polyploidization, which is recognized as a relevant evolutionary force, playing an important role in plant adaptation and speciation [4]. Artificially induced polyploidy is a useful tool in plant breeding that usually enhances species yield and adaptability [1].

Polyploidy is a highly appreciated condition among plant breeders because it confers a broad spectrum of traits that enhance plant fitness. Artificial polyploidization has been used in plant breeding to increase phenotypic variation, frequently influencing organ size, stomatal traits, photosynthetic efficiency, specialized metabolism, and, in some species, agronomic performance. Common phenotypic modifications observed in polyploid plants include increased leaf and fruit size [5,6,7], improved photosynthetic performance [8,9], greater accumulation of secondary metabolites [3,10], positive impacts on rhizobia mutualistic interactions [11], and enlarged stomata accompanied by reduced stomatal density [12,13]. Stomatal modifications are of particular interest for agriculture under current and future climate change scenarios, as stomatal size and density together determine the stomatal pore area index (SPI), which provides an anatomical estimate of the potential epidermal pore area available for gas exchange. A lower SPI may restrict potential stomatal conductance and transpiration water loss, thereby contributing to water conservation under drought [14]. In common bean, experimental evidence has associated drought resistance with water-saving stomatal responses, including reduced stomatal density and earlier stomatal closure under water limitation [15], making polyploidization a potential strategy to modify leaf diffusive properties and, consequently, improve plant water-use efficiency under drought conditions [16].

Artificial polyploidy induction can be achieved through chemical, physical, or biological approaches. Chemical methods are the most widely used and rely on reagents that disrupt microtubule formation and chromosome segregation during cell division before cytokinesis, resulting in the formation of polyploid cells [17]. Colchicine is the most used reagent. The efficiency of colchicine-induced polyploidization depends on several factors, such as reagent concentration, time of exposure, explant type, and the intrinsic sensitivity of the plant species [18].

Legume species have also been targets of induced polyploidization efforts, including cowpea and soybean, among others [19,20]. These studies have reported changes in stomatal traits and contrasting effects on yield [21].

Common bean (*Phaseolus vulgaris*), the most important legume for direct human consumption, is a species with high sensitivity to drought, with yield losses that can approach 100% under severe water limitation [15,16]. Because colchicine acts on dividing cells, directly treated C0 plants may contain different ploidy levels among tissues or cell lineages. Consequently, the detection of tetraploids in vegetative tissue does not necessarily demonstrate that chromosome doubling occurred in the reproductive cell lineage or that the tetraploid condition will be transmitted through the gametes. For breeding purposes, evaluating C1 progeny is, therefore, essential to determine whether viable tetraploid offspring can be recovered and used to establish stable breeding material [22]. In common bean, however, it remains uncertain whether colchicine-induced polyploidization can consistently generate stable tetraploids with stomatal and physiological phenotypes that are both agronomically favorable and transmissible to the next generation [22].

Here, we induced polyploidy in common bean and evaluated whether confirmed tetraploid lines exhibited coordinated changes in stomatal anatomy, gas exchange, pigment composition, and antioxidant-related traits. We also analyzed the extent to which induced ploidy was transmitted to the next generation.

## 2. Results

### 2.1. Colchicine Induces Tetraploid and Mixoploid Plants with Variable Efficiency

**Figure 1:**
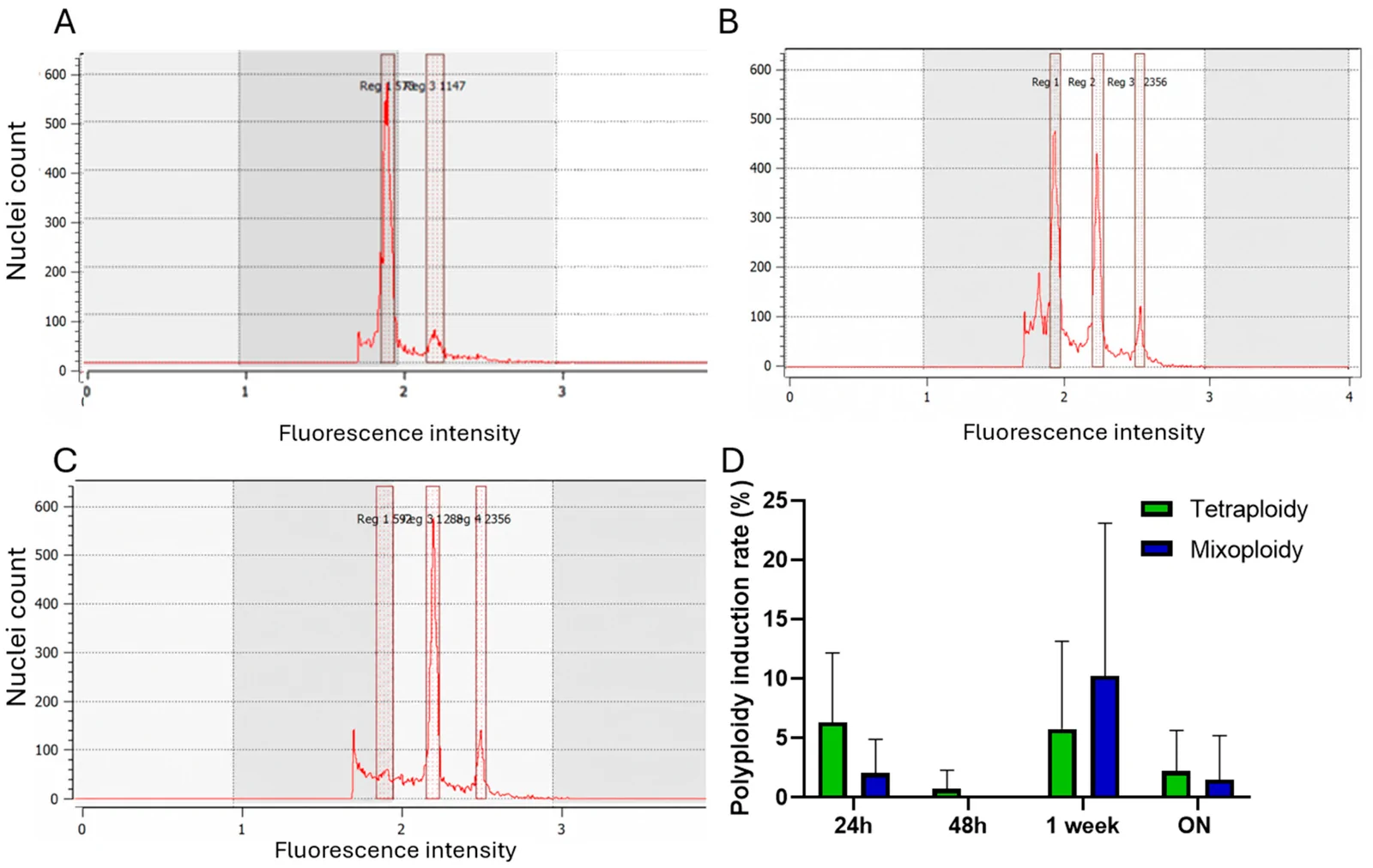
Polyploidy determination**.** (**A**–**C**): The representative histograms of nuclei quantification. (**A**) The diploid control plant, (**B**) the mixoploid plant, (**C**) the tetraploid plant, and (**D**) the polyploidy induction rate. The green bars indicate the tetraploid induction rate, and the blue bars indicate the mixoploidy induction rate. The induction rates were calculated as the number of polyploid plants relative to the number of germinated seeds per assay. No significant differences were observed among the treatments (One-way ANOVA, *p* ≤ 0.05).

The colchicine treatments successfully generated confirmed tetraploid C0 plants in common bean across all tested exposure regimes. A total of 15 tetraploid plants were recovered: two following overnight soaking, seven after 24 h imbibition, one after 48 h imbibition, and five after 1-week imbibition. Mixoploid individuals were also identified in addition to tetraploids. The representative flow-cytometry histograms for diploid, mixoploid, and tetraploid plants are presented in Figure 1A–C. While no significant differences in the induction rate were observed among the colchicine treatments, induction frequencies varied substantially among independent assays. Tetraploid induction rates exhibited high between-assay variability, with coefficients of variation of 91.9%, 223.6%, 127.2%, and 154.9% for the 24 h, 48 h, 1-week, and overnight treatments, respectively. Similarly, mixoploid induction rates showed marked variability among replicates, with coefficients of variation of 137.6%, 0%, 125.5%, and 244.9% for the 24 h, 48 h, 1-week, and overnight treatments, respectively. These results indicate a highly heterogeneous response of common bean seedlings to colchicine exposure, even under identical treatment conditions.

### 2.2. Tetraploidy in Phaseolus vulgaris Did Not Consistently Reduce Stomatal Pore Area Index

**Figure 2:**
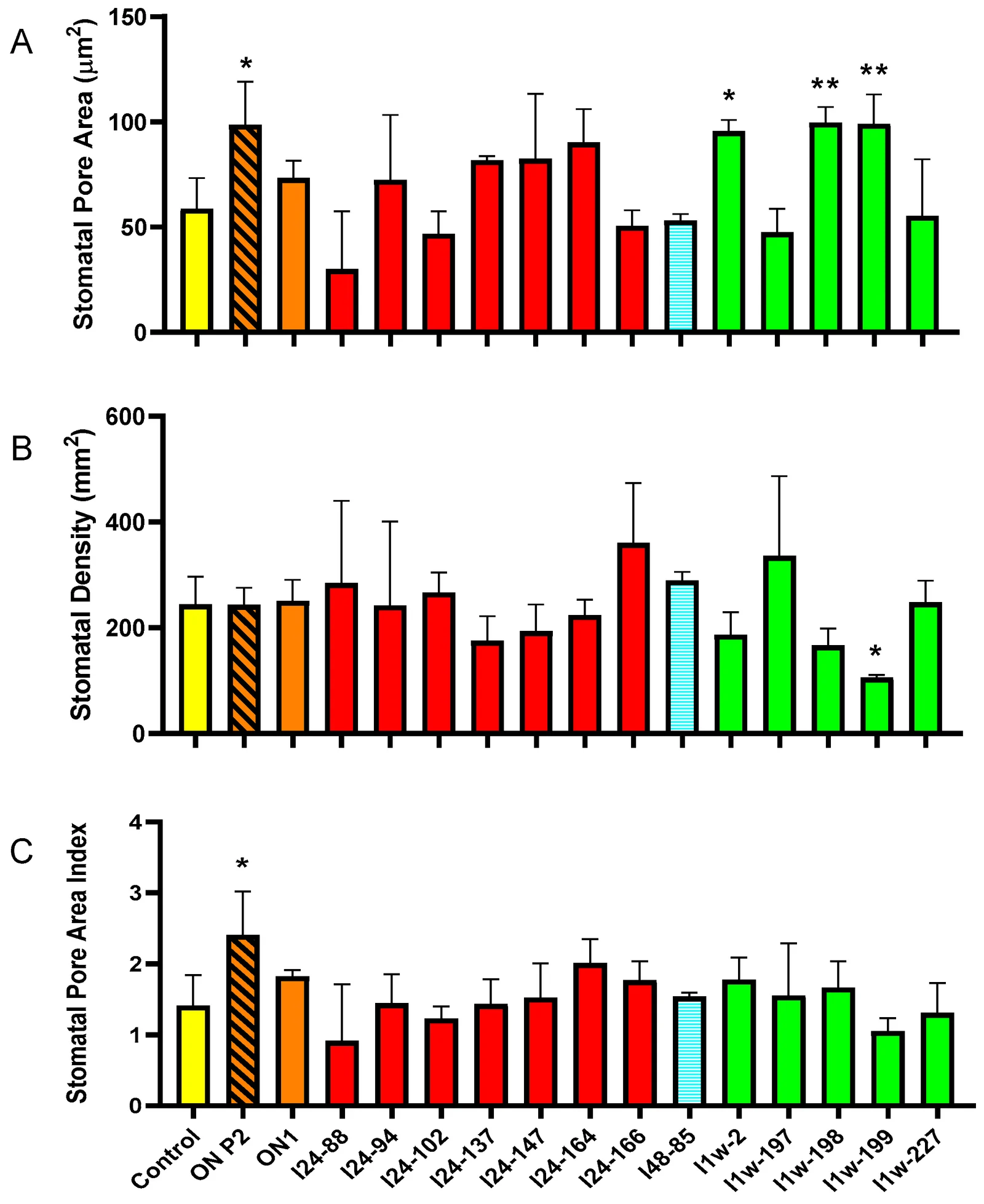
Stomatal anatomical parameters**.** (**A**) SPA, calculated by approximating stomatal pore measurements to an ellipse; (**B**) SD, calculated as the number of stomata per mm^2^; and (**C**) the SPI. The different colors and patterns represent the colchicine application treatments. Yellow: the control without colchicine; striped, orange: overnight colchicine soaking of C1; orange: overnight colchicine soaking of C0; red: 24 h colchicine imbibition; light blue: 48 h colchicine imbibition; and green: 1-week colchicine imbibition. Statistical differences are indicated with asterisks (* *p* ≤ 0.05, ** *p* ≤ 0.01).

Stomatal anatomical profiling of the confirmed tetraploid lines demonstrated only partial and line-specific shifts in the expected direction. The stomatal pore area (SPA) was significantly higher in four tetraploid lines (ON P2, I1w-2, I1w-198, and I1w-199) (Figure 2A), whereas stomatal density (SD) was significantly reduced only in I1w-199 compared to the control (Figure 2B). However, these alterations did not result in a consistent reduction in the SPI, which was the main anatomical target of the induction strategy. The stomatal pore index remained unchanged in most tetraploid lines and was significantly higher in ON P2 than in the control (Figure 2C). Therefore, induced tetraploidy did not consistently produce the stomatal phenotype expected to reduce the effective diffusive surface at the leaf level.

### 2.3. Gas-Exchange Traits Were Highly Heterogeneous Among Tetraploid Lines

Gas-exchange profiling revealed a physiological trade-off between carbon assimilation and water-use efficiency among the tetraploid lines, rather than a uniform improvement in photosynthetic performance. Relative to the control, several lines showed a higher net photosynthesis rate (A), particularly I48-85, I1w-197, I1w-198, and I1w-199; in most cases, this increase in A co-occurred with higher stomatal conductance (g_s_) and, in some lines, a higher substomatal carbon concentration (C_i_). Transpiration (E) also tended to be higher in several tetraploid lines, reaching statistical significance only in I1w-198. Critically, this coordinated increase in A, g_s_, and E was consistently accompanied by a decline in intrinsic water-use efficiency (WUE_i_), indicating that the higher carbon assimilation observed in several tetraploid lines was achieved at the expense of increased diffusive water loss rather than through an improvement in water-use efficiency. Overall, these results point to a recurrent physiological trade-off among colchicine-derived tetraploid lines, in which enhanced gas-exchange performance was systematically linked to reduced water-use efficiency, rather than to a uniform photosynthetic improvement (Table 1).

### 2.4. Pigment Composition Changed Only in a Subset of Tetraploid Lines

Changes in pigment composition were limited and line-specific. Significant changes in chlorophyll *a* occurred only in I24-88 and I1w-197; both lines also exhibited higher chlorophyll *b.* Additional increases in chlorophyll b were detected in I24-137, I24-164, and I48-85. Carotenoid responses were generally inconsistent; however, I24-88 demonstrated a coordinated increase in lutein, neoxanthin, and violaxanthin. Antheraxanthin levels remained unchanged among the evaluated tetraploid lines. Taken together, these results suggest that induced tetraploidy did not broadly affect pigment composition across all lines, although selected tetraploids exhibited coordinated increases in chlorophylls and specific xanthophylls (Table 2).

### 2.5. Oxidative and Antioxidant Traits Varied Among Lines with No Consistent Stress Signature

Oxidative and antioxidant characteristics varied among the tetraploid lines. TBARS values did not consistently increase in the tetraploid lines compared with the control, indicating that lipid peroxidation did not show a consistent trend after polyploid induction. In contrast, antioxidant-related parameters showed line-specific changes. Notable differences in radical scavenging activity were observed among the lines, and the total phenolic concentration was higher in selected cases, particularly ON P2 and I1w-198. Under the non-stress growth conditions and developmental stage evaluated, these measurements did not reveal a consistent lipid peroxidation- or antioxidant-related response among the tetraploid lines. Instead, TBARS, radical-scavenging capacity, and the total phenolic content exhibited line-specific variation (Table 3).

### 2.6. Multivariate Analyses Resolved Coordinated Phenotypic Structure Among Tetraploid Lines

#### 2.6.1. Correlation Among Variables

**Figure 3:**
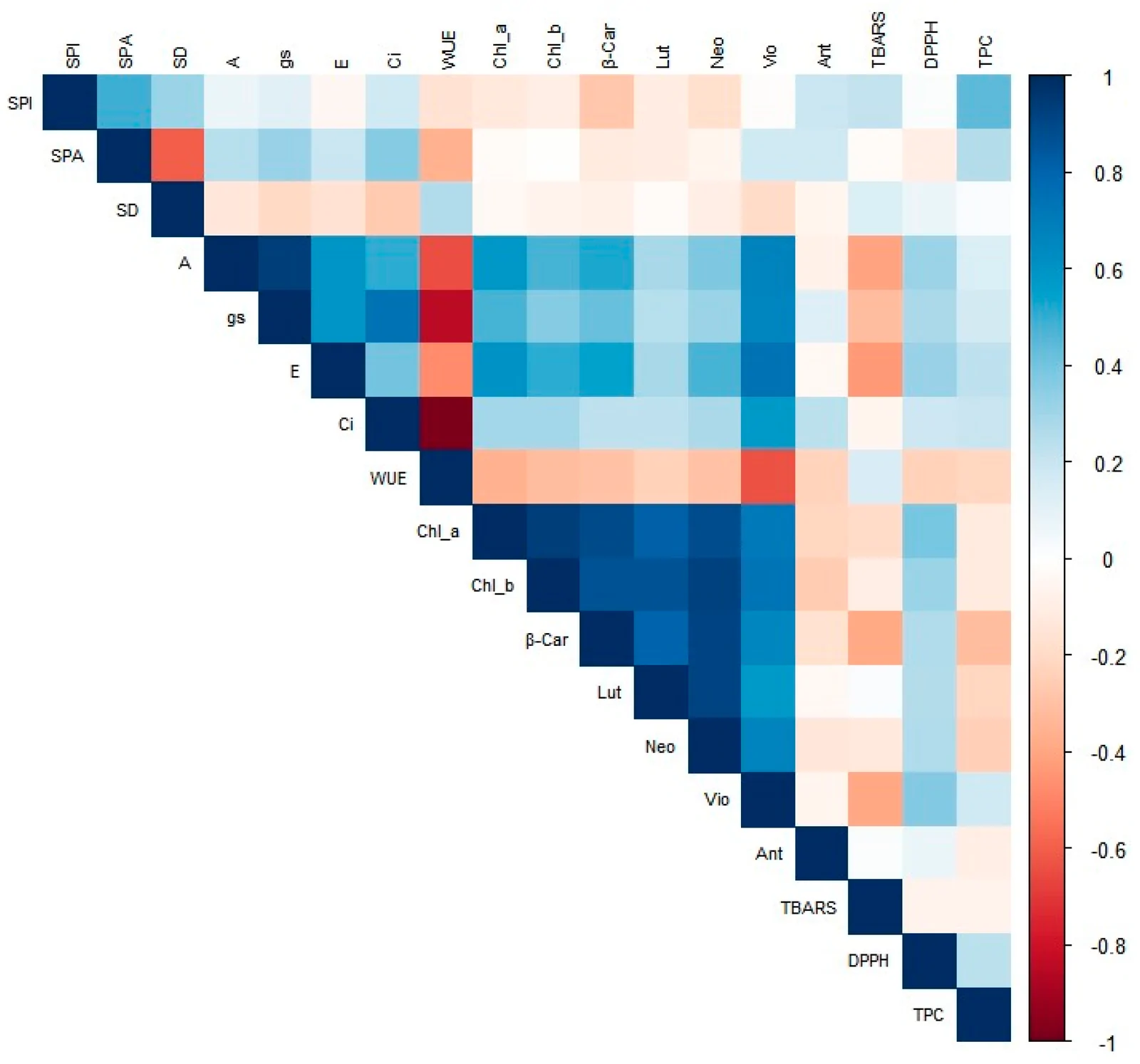
A correlation matrix of physiological, stomatal, pigment, and oxidative stress–related traits among the bean genotypes. The heatmap displays the Pearson correlation coefficients among the stomatal anatomical parameters (SPI, SPA, and SD), gas-exchange traits (A, g_s_, E, C_i_, and WUE_i_), photosynthetic pigment composition (Chl *a*, chlorophyll *a*; Chl *b*, chlorophyll *b*; β-Car, β-carotene; Lut, lutein; Neo, neoxanthin; Vio, violaxanthin; and Ant, antheraxanthin), and oxidative/antioxidant indicators (TBARS, DPPH, and TPC (total phenolic content)). The color intensity represents the correlation strength (blue = positive, red = negative), with darker tones indicating stronger associations. Only the upper triangular portion of the matrix is shown.

Correlation analysis showed coordinated associations among stomatal, physiological, pigment-related, and oxidative/antioxidant variables. By contrast, WUE_i_ was negatively associated with g_s_, E, and C_i_. Stomatal anatomical traits showed weaker relationships with gas-exchange variables, whereas several pigment-related traits were positively associated with A, g_s_, and E. TBARSs tended to be negatively associated with photosynthetic and pigment variables, while antioxidant-related variables showed line-dependent associations within the integrated trait network (Figure 3).

A, g_s_, and E were strongly and positively correlated (r > 0.70) and were also positively associated with Ci, indicating coordinated regulation of gas exchange. In contrast, WUE_i_ was negatively correlated with g_s_, E, and C_i_, reflecting the trade-off between carbon assimilation and water loss. Stomatal anatomical traits showed moderate correlations with photosynthetic variables, suggesting that anatomy contributes to, but does not strictly determine, gas-exchange performance.

Photosynthetic pigments were positively correlated with A, g_s_, and E (r > 0.60), with xanthophyll cycle carotenoids showing particularly strong associations with photosynthetic activity. Oxidative stress markers were also integrated within this network: TBARSs showed negative correlations with photosynthesis and pigment content, whereas antioxidant capacity indicators (DPPH and total phenols) were negatively correlated with TBARSs and positively correlated with photosynthetic and pigment traits.

#### 2.6.2. Principal Component Analysis

**Figure 4:**
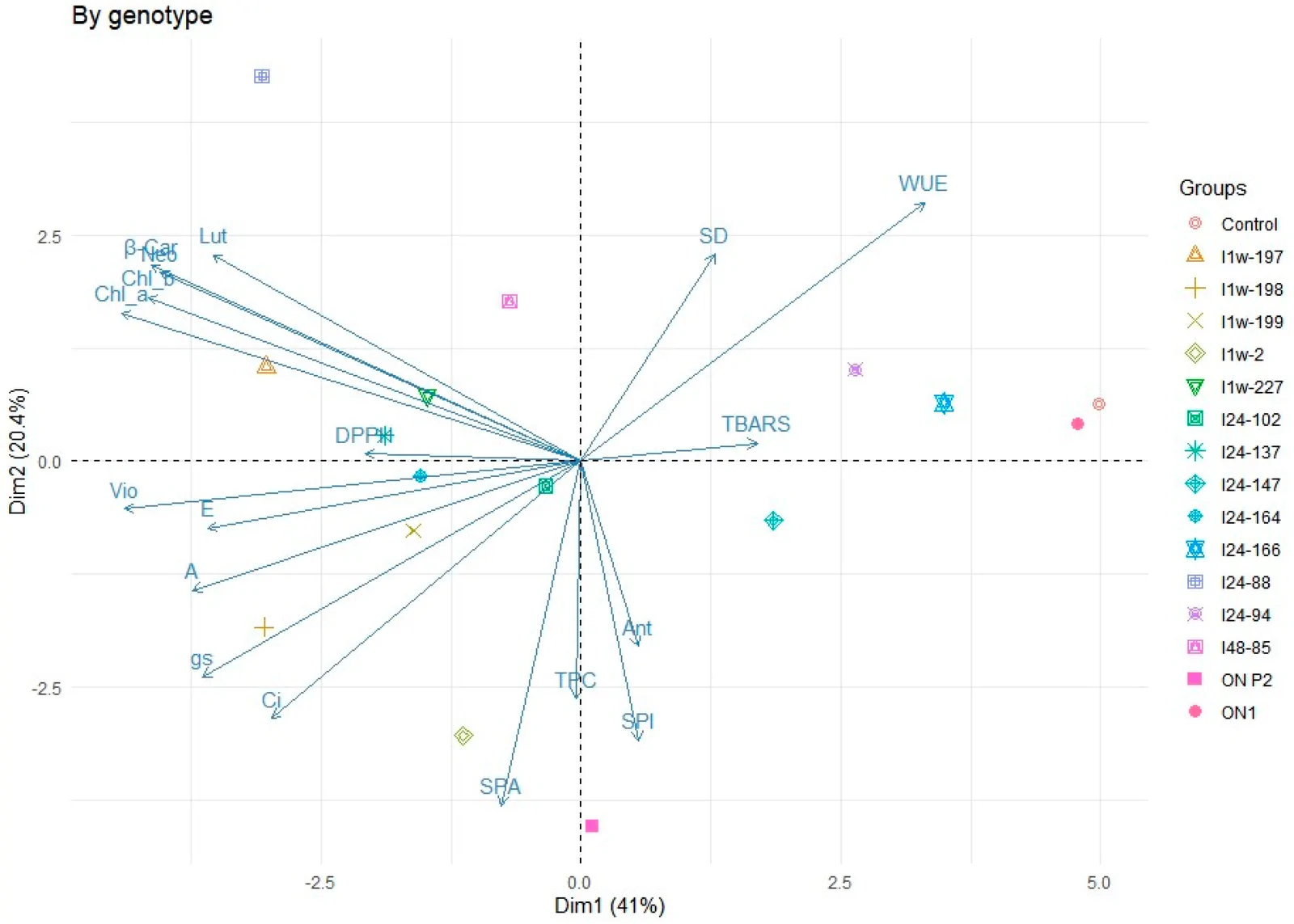
Principal component analysis of the mean stomatal, gas-exchange, pigment, and oxidative/antioxidant traits among the bean genotypes. The biplot shows the position of each genotype in the multivariate space, defined by the first two principal components (PC1 and PC2), explaining 41.0% and 20.4% of total variance, respectively. The arrows represent the standardized loadings of stomatal anatomical traits (SPI, SPA, and SD), gas-exchange variables [A, g_s_, E, C_i_, and WUE_i_) photosynthetic pigments (Chl *a*, Chl *b*, β-Car, Lut, Neo, Vio, and Ant), and oxidative/antioxidant indicators (TBARS, DPPH, and TPC).

Principal component analysis (PCA) further elucidated the multivariate structure of the dataset (Figure 4). The first two principal components explained 61.4% of the total variance, with PC1 accounting for 41.0% and PC2 for 20.4%. PC1 primarily separated lines according to coordinated variation in gas-exchange performance, pigment content, and oxidative/antioxidant traits, whereas PC2 captured additional variation related to pigment composition, stomatal traits, and redox-related variables. Together, these axes differentiated tetraploid lines with contrasting integrated phenotypic profiles, highlighting the broad heterogeneity generated by colchicine treatment.

#### 2.6.3. Hierarchical Clustering of Tetraploid Genotypes

**Figure 5:**
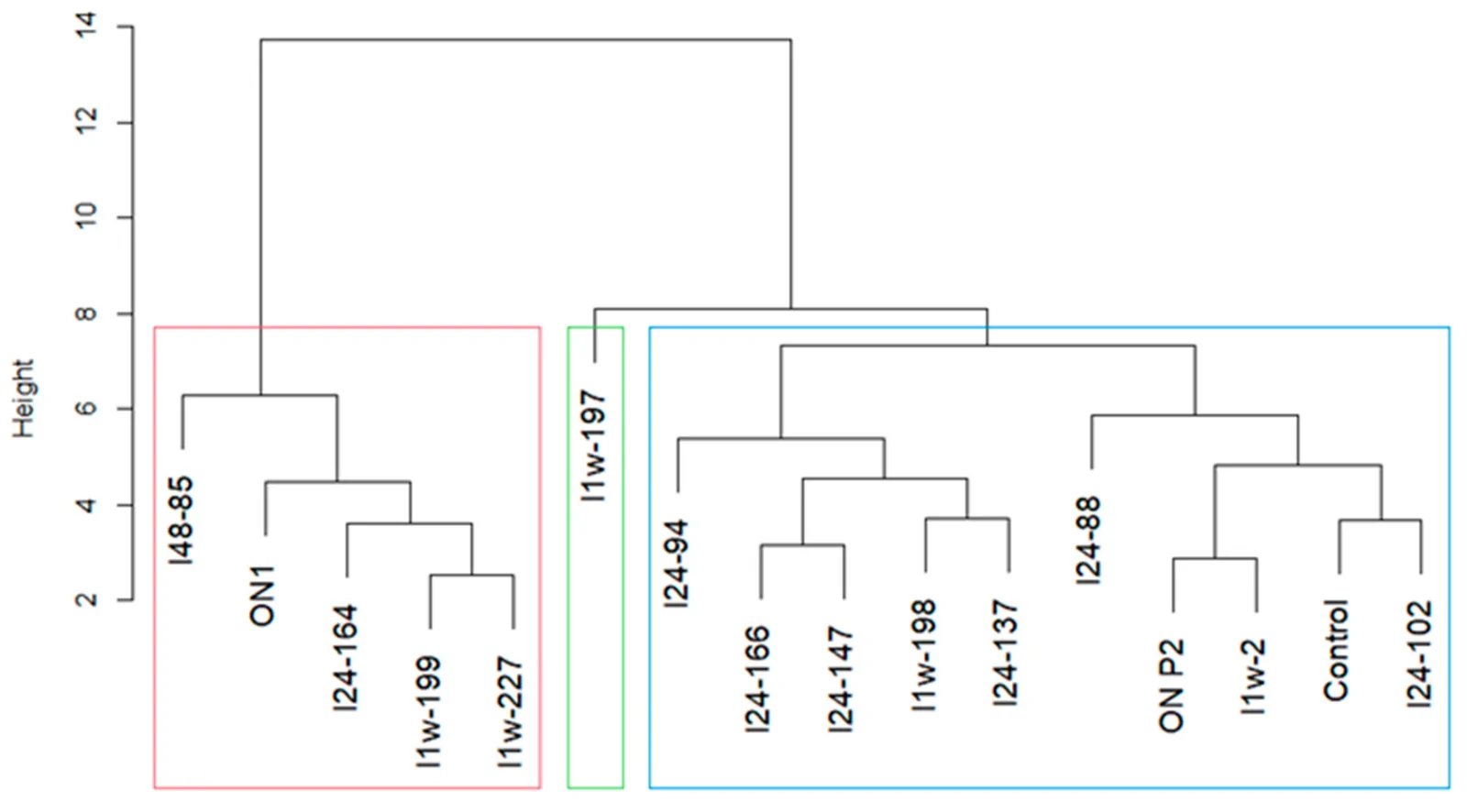
Hierarchical clustering of the bean genotypes based on multivariate physiological performance. A dendrogram, constructed from Euclidean distances among the genotypes using the standardized (z-score) mean values of stomatal anatomical traits (SPI, Ap, and SD), gas-exchange variables (A, g_s_, E, C_i_, and WUE_i_), pigment-related traits (Chl *a*, Chl *b*, β-Car, Lut, Neo, Vio, and Ant), and oxidative/antioxidant indicators (TBARS, DPPH, and TPC), and clustered using Ward’s linkage. Cutting the tree at k = 3 reveals three major multivariate groups: (i) a cluster comprising I48-85, ON1, I24-164, I1w-199, and I1w-227 (left, red box), indicating a shared physiological signature among the integrated trait set; (ii) a distinct intermediate branch formed by I1w-197 (central, green box), highlighting a unique overall profile relative to the remaining genotypes; and (iii) a second major cluster, including I24-94, I24-166, I24-147, I1w-198, I24-137, I24-88, ON P2, I1w-2, the control, and I24-102 (right, blue box), reflecting broader similarity among these genotypes in their combined stomatal, photosynthetic, pigment, and oxidative traits.

Hierarchical clustering analysis based on the standardized mean values of stomatal anatomical gas traits, gas-exchange parameters, pigment composition, and oxidative and antioxidant indicators revealed a well-defined multivariate structure among the bean genotypes (Figure 5). The dendrogram showed a major separation at a height of approximately h ≈ 13−14, indicating pronounced divergence among the main genotype groups.

A first cluster, separated from the remaining genotypes at h ≈ 7.5, comprised I48-85, ON1, I24-164, I1w-199, and I1w-227. These genotypes were characterized by negative or moderately low PC1 scores, ranging approximately from −1.61 to −0.34. Within this cluster, I48-85 and ON1 exhibited the closest multivariate proximity, clustering together at a height below h ≈ 4.

A second cluster was represented exclusively by genotype I1w-197, which formed an isolated branch and diverged from the remaining genotypes at an intermediate height (h ≈ 6.5–7). This genotype displayed a distinct multivariate profile relative to the other groups.

The third and largest cluster included I24-94, I24-166, I24-147, I1w-198, I24-137, I24-88, ON P2, I1w-2, I24-102, and the control plants and consolidated at approximately h ≈ 8. Within this group, a clear internal substructure was observed, consistent with the gradient defined by PC1. Genotypes such as the control, I24-166, and I24-94 clustered closely together, whereas I1w-198 and I1w-2 occupied more distant branches within the same cluster, indicating increased internal heterogeneity.

Overall, hierarchical clustering confirmed the organization of the genotypes into coherent multivariate groups, in agreement with the structure observed in the PCA and the ranking based on PC1 scores.

#### 2.6.4. Ranking of Bean Genotypes According to a Gas-Exchange Performance Index

**Figure 6:**
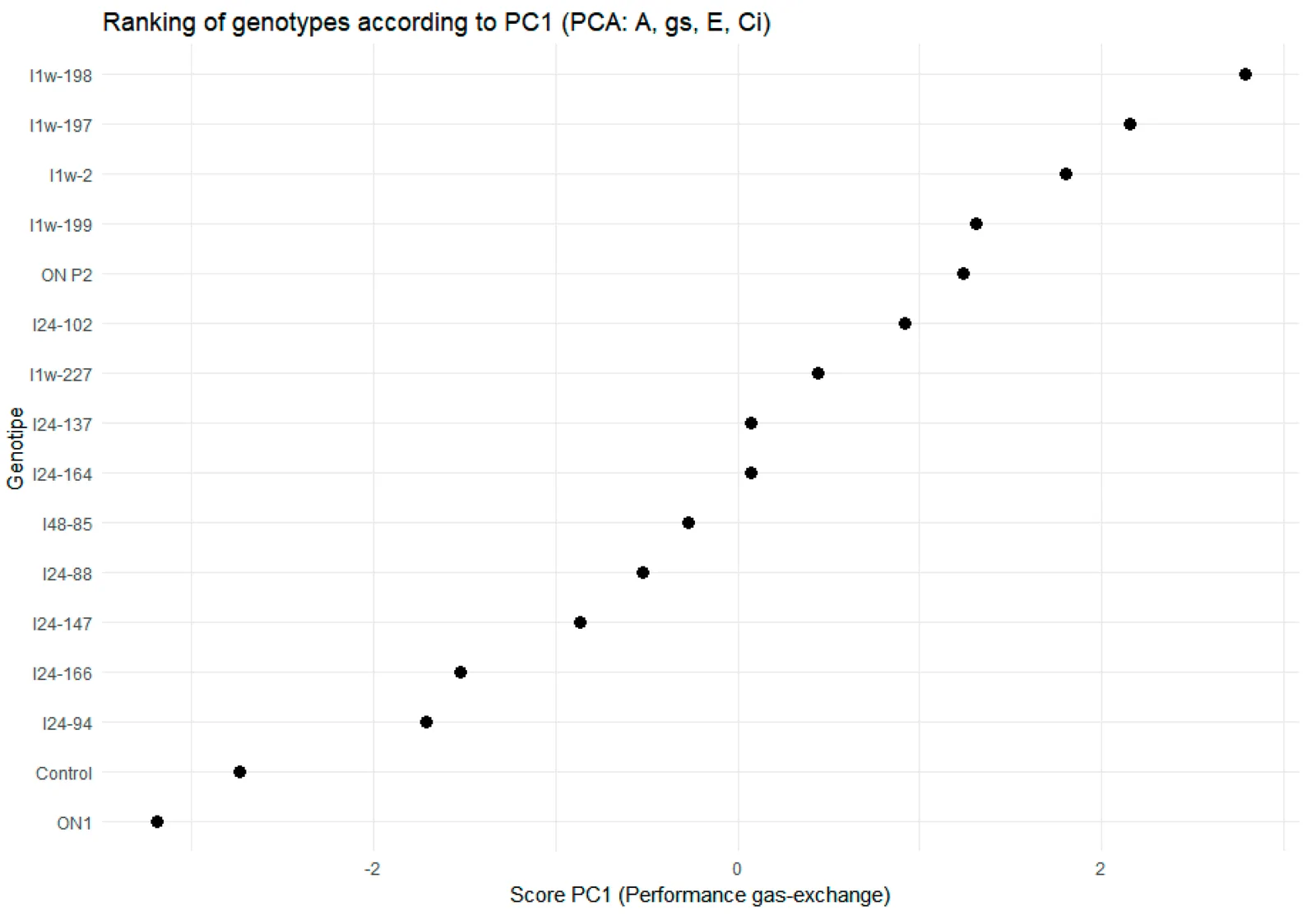
The ranking of the bean genotypes according to a gas-exchange performance index (PC1 from the PCA of trait means). The plot shows the ordering of the genotypes based on their PC1 scores, derived from the PCA and computed using the genotype means of gas-exchange traits (A; g_s_; E; and C_i_). Higher PC1 values indicate the genotypes with overall stronger gas-exchange performance among these coordinated traits. Genotypes I1w-198, I1w-197, and I1w-2 ranked highest, whereas ON1 and the control occupied the lowest positions, reflecting comparatively reduced integrated gas-exchange performance.

A PCA restricted to gas-exchange traits (A, g_s_, E, and C_i_) was used to derive an integrated gas-exchange performance index from the first principal component. Based on this ranking, I1w-198, I1w-197, and I1w-2 showed the highest PC1 scores, followed by I1w-199 and ON P2. In contrast, ON1 and the control occupied the lowest positions (Figure 6). This analysis indicates that several tetraploid lines displayed comparatively stronger integrated gas-exchange profiles than the control, although this trend was not uniformly associated with the target stomatal phenotype.

### 2.7. Tetraploidy Inheritance

Fifteen tetraploid plants obtained from the different induction treatments were allowed to set seed. Eleven of these plants produced seeds, and their progeny were evaluated by flow cytometry. Table 4 represents the total number of seeds per plant and the resulting ploidy of the evaluated C1 plants. Lines ON1, I24-88, I24-102, and I24-137 neither flowered nor produced seeds. Most C1 individuals analyzed were identified as diploid, indicating limited transmission of tetraploidy to the next generation. A single exception was observed in a preliminary assay, where one tetraploid C0 plant produced a single seed. The resulting C1 individual (ON P2) was also tetraploid and was included in the physiological analyses; however, this plant did not produce progeny.

## 3. Discussion

Colchicine application treatments successfully resulted in the generation of tetraploid plants. The tetraploid induction rates obtained in our study are comparable to those previously reported for 0.1% colchicine application to seeds in ajowan (3.8–7.6%) [23] and 16.7% in *Platanus acerifolia* [24]. The duration of the treatment did not produce a statistically significant effect on the polyploidy induction rate. However, a trend toward increased induction was observed with one-week treatment. This result aligns with the results from Sadat-Noori et al. [23], who analyzed treatments ranging from 6 to 48 h and reported the highest polyploidy induction rate at 24 h.

No significant differences in the mixoploidy induction rate were observed between treatments. Reports of mixoploidy in colchicine-induced systems remain relatively scarce, although previous studies have described variability in response to the colchicine concentration and exposure duration [25,26,27]. The observed high variability in mixoploid frequency in this study further supports the conclusion that colchicine effects are spatially and temporally heterogeneous within developing tissues, leading to the formation of chimeric plants rather than stable, whole-organism polyploids.

An important consideration when interpreting the phenotypic data in this study is that each tetraploid line originated from a single independent colchicine-derived C0 plant. Thus, individual lines do not represent biological replicates of a common tetraploid genotype. Phenotypic differences restricted to individual lines are, therefore, interpreted here as line-specific or event-specific responses. Only patterns consistently observed among multiple independently derived tetraploid plants are considered potential common responses associated with chromosome doubling in cv. ‘Zorzal’.

Modification of stomatal size and density is a key target for improving drought tolerance through induced polyploidy. Larger, naturally occurring genome sizes are correlated with increased stomata size and reduced stomatal density among angiosperms [28,29]. Several studies have demonstrated that chemically induced polyploidy results in decreased stomatal density and increased stomatal size [26,30,31]. Nevertheless, other research has challenged this association by reporting neopolyploid lines without significant anatomical changes [25,32].

In our results, among the colchicine-treated plants, four lines exhibited larger stomata, resulting in an increased stomatal pore area, while only one line showed a significant reduction in stomatal density. However, these anatomical changes did not correspond to consistent alterations in the SPI, which remained largely unchanged among lines and even increased in ON P2 compared to the control. Because the SPI is calculated as the product of the stomatal pore area and stomatal density, its response depends on the magnitude and direction of change in both components. In I1w-199, the reduction in stomatal density counterbalanced the increase in pore area, resulting in no significant change in the SPI. Conversely, ON P2 showed an increased pore area without a compensatory reduction in density and consequently exhibited a significantly higher SPI. In the other lines with an increased pore area, stomatal density did not show a coordinated, statistically significant response, and variability in both components prevented a consistent change in their product. These results challenge the widely assumed causal relationship between genome duplication and the presence of larger stomata and lower stomatal density [28,33,34], suggesting that induced polyploidy does not reliably produce coordinated anatomical outcomes in common bean cv. ‘Zorzal’. Despite the limited consistency observed in stomatal anatomical traits, a detailed analysis of physiological parameters was conducted to assess the potential functional consequences of polyploidy. This approach allowed us to evaluate both anatomical variation and the emergence of eco-physiological phenotypes.

In our study, four tetraploid lines exhibited significantly increased stomatal pore areas, whereas stomatal density was significantly reduced only in I1w-199. Previous studies of induced polyploids have frequently reported the characteristic combination of larger stomata and lower stomatal density [26,30,31]. However, these opposing anatomical responses do not necessarily produce a predictable change in the SPI because the resulting index depends on their proportional balance. Thus, the relatively stable SPI observed in most lines does not contradict the occurrence of enlarged stomata; rather, it reflects the absence of a uniform and coordinated scaling response between the stomatal pore area and density. These results indicate that chromosome doubling did not reliably produce the coordinated anatomical phenotype required to reduce the potential stomatal pore area at the leaf level in *P. vulgaris* cv. ‘Zorzal’.”

Gas-exchange traits (A, g_s_, and E) exhibited strong positive correlations (r > 0.70), indicating that these variables covaried among the evaluated lines. These statistical associations are consistent with the established physiological role of stomatal conductance in regulating CO_2_ diffusion and transpiration water loss [35,36], as further supported by the regression models showing that g_s_ explains a substantial proportion of the variation in A, including a partially non-linear relationship. From a functional perspective, this behavior reflects a classical diffusion-driven system in which CO_2_ supply to the chloroplasts is primarily governed by stomatal aperture [37].

C_i_ was also positively associated with g_s_ and A [38]. Although this relationship is consistent with differences in CO_2_ diffusion among lines, C_i_ results from the balance between CO_2_ supply and photosynthetic consumption and, therefore, cannot be attributed to stomatal conductance alone. Likewise, the present data do not allow us to determine whether changes in substomatal anatomy contributed to variation in C_i_. Few studies have examined C_i_ in induced polyploids. Domínguez-Delgado et al. [39] reported no changes in C_i_ in induced polyploids of *Dianthus broteri*, and Zhang et al. [40] reported a decrease in tetra- and hexaploid *Brassica rapa* in comparison to diploid plants because of a decrease in photosynthesis. This observed heterogeneity among induced polyploids suggests a lack of consistency between stomatal anatomical traits and ploidy level. 

A physiological trade-off between carbon gain and water conservation was identified. The negative correlations between WUE_i_ and g_s_, E, and C_i_ indicate that genotypes that maximize photosynthesis exhibit greater water loss. Principal component analysis (PCA) further reinforced this trade-off, with PC1 capturing a dominant gradient separating the high gas-exchange genotypes from those with higher WUE_i_ and lower transpiration fluxes.

This pattern is consistent with the established continuum between anisohydric and isohydric strategies [41,42]. Genotypes with high PC1 scores (e.g., I1w-198, I1w-197, and I1w-2) demonstrate more anisohydric behavior by maintaining higher stomatal conductance and carbon assimilation with less restrictive control of water loss. In contrast, genotypes including the control and ON1 exhibit a more conservative, isohydric-like strategy. Notably, these contrasting strategies emerged independently of any consistent anatomical or ploidy-driven pattern, which further supports the hypothesis that polyploidization increases physiological variability rather than directing it toward a specific adaptive outcome [43]. The high variability observed among independent assays and among the recovered tetraploid plants probably resulted from several non-exclusive sources. Individual seedlings may have differed in physiological condition, developmental stage, seed-coat permeability, colchicine uptake, mitotic activity, and intrinsic sensitivity to the antimitotic treatment. Although the colchicine concentration, exposure duration, seed number, temperature, and post-treatment conditions were standardized, minor procedural differences in seed imbibition, contact with the colchicine solution, germination, and recovery may also have contributed to the between-assay variation. Furthermore, because each tetraploid plant originated from an independent chromosome doubling event, part of the subsequent phenotypic heterogeneity may reflect event-specific biological effects, including altered gene dosage, genomic or epigenetic responses, and differences in the tissue distribution of doubled cells. Colchicine itself may also produce phenotypic effects that are independent of chromosome doubling [18]. Because these potential sources were not experimentally partitioned, their relative contributions cannot be determined from the present study.

Photosynthetic pigments showed strong positive associations with gas-exchange traits (r > 0.6), indicating that genotypes with higher carbon assimilation also possess a more developed photosynthetic apparatus. The coordinated increase in chlorophylls and carotenoids suggests that enhanced light capture is accompanied by increased photochemical capacity. Carotenoids involved with the xanthophyll cycle were positively correlated with photosynthetic activity, underscoring their role in photoprotection [44]. However, these coordinated responses were limited to specific lines and did not constitute a general effect of polyploidization induction. The absence of a shared directional response indicates that changes in chlorophyll and carotenoid contents were specific to each line or event-dependent rather than a general physiological consequence of chromosome doubling in *P. vulgaris* cv. ‘Zorzal’. These differences may reflect variation in pigment metabolism or physiological state among independent induction events, but the underlying mechanisms were not directly evaluated.

The observed relationship between oxidative stress markers and physiological traits underscores the integrative nature of the system. TBARS, an indicator of lipid peroxidation [45], was negatively correlated with both photosynthesis and pigment concentration, whereas antioxidant capacity, as measured by DPPH and phenolics, exhibited an opposite pattern. This pattern was dependent on the specific plant genotype, suggesting that redox regulation is not uniformly influenced by polyploidy but instead varies among independently derived genotypes. This finding is particularly relevant in the context of polyploidy, as increased metabolic activity could potentially enhance reactive oxygen species (ROS) production [46]. The results indicate that successful genotypes may compensate for this by upregulating antioxidant defenses, achieving a stable physiological state.

The PCA and hierarchical clustering analyses revealed that genotypes are organized along coordinated multivariate gradients rather than isolated trait differences. PC1 emerged as a robust descriptor of gas-exchange performance, while PC2 captured variation related to pigment composition and oxidative balance. Together, these axes define a multidimensional eco-physiological space where genotypes occupy distinct functional niches. This multivariate structure indicates that polyploidization increases the dimensionality of phenotypic variation, effectively expanding the accessible trait space rather than shifting it in a consistent direction [47].

A key insight from the ranking analysis indicates that high-performing genotypes achieve an optimal balance among traits rather than maximizing individual parameters. This suggests that polyploidization does not inherently improve performance but may generate rare genotypes with favorable integrated phenotypes through stochastic variation. This concept of optimization is, therefore, critical for interpreting polyploid performance.

A critical limitation of colchicine-induced polyploidy in common bean is the lack of ploidy stability across generations. Despite the successful recovery of plants classified as tetraploid, based on flow cytometry analysis of C0 leaf tissue, most of the evaluated C1 progeny were diploid, indicating limited transmission of the tetraploid condition to the subsequent generation.

The mechanisms underlying the predominance of diploid progeny observed in this study remain uncertain, and several non-exclusive hypotheses may be considered. One possible explanation is meiotic instability in induced tetraploids, whereby irregular chromosome pairing and segregation could generate unbalanced gametes and reduce the transmission of tetraploid chromosome complements to the next generation, as described for colchicine-induced auto-octoploids of Persicaria tinctoria [48], or from a somatic reversion of ploidy level in the branches where flowering occurred. In octoploid *P. tinctoria* plants, fertility was reported to be extremely low, eventually leading to lineage cessation. This reduction in fertility was proposed to result from irregular meiotic chromosome behavior, where multivalent configurations, such as quadrivalents, trivalents, and univalents, formed instead of exclusively bivalent pairing, ultimately causing unbalanced chromosomal segregation and the production of aneuploid gametes [49]. Similar meiotic instability could potentially contribute to the reduced fertility and predominance of diploid progeny observed in our tetraploid common bean lines.

Another hypothesis is that undetected chimerism or tissue-specific ploidy instability may have contributed to the observed predominance of diploid progeny if reproductive tissues originated from diploid cell sectors not represented in the leaf tissue used for ploidy determination. The tetraploid plants reported here were identified by flow cytometry using leaf tissue, and the histograms shown in Figure 1C are consistent with tetraploids in the sampled tissue. However, because reproductive tissues were not directly analyzed, the possibility of tissue-specific differences in ploidy composition cannot be completely excluded.

A similar phenomenon was reported in induced autotetraploid *Zea mays*, in which surviving plants maintained the altered ploidy level until the seventh-leaf stage, prior to anthesis, before subsequently reverting to the diploid state [50]. Interestingly, re-diploidized maize plants were morphologically similar to diploids for most evaluated traits, suggesting that genome downsizing may occur without major phenotypic disruption. These observations highlight the possibility that induced polyploidization in some species may represent a transient genomic condition rather than a stable heritable state.

Our results can be contrasted directly with the excellent works on polyploidy induction in legumes from Biswas and Bhattacharyya [20,22,51]. In these studies, colchicine polyploidy induction was performed successfully in three legumes: *Cyamopsis psoraloides*, *Glycine max*, and *P. vulgaris*. A reduced fertility rate was observed for *C. psoraloides* and *G. max*, explained by altered pollen structure and increased sterility. However, in the case of *P. vulgaris*, a second generation of colchicine-induced tetraploids was not achieved at all [22], matching our results. Although pollen anatomy was not evaluated, reduced seed production in our tetraploid lines is consistent with previously reported reproductive instability in induced legume polyploids. Autogamous flowers [52] from tetraploid plants could fail during autopollination due to altered pollen anatomy [20,22,51,53] or pollen germination, as previously reported for neopolyploids [54]. Interallelic interactions, which have been described in autopolyploid *Petunia hybrida* [55], may additionally contribute to reproductive instability and reduced fertility in induced polyploids.

Because neither meiotic chromosome behavior nor whole-plant ploidy composition was directly evaluated, the relative contribution of the previously proposed mechanisms remains unresolved. Future studies should combine chromosome counting, meiotic chromosome analyses, tissue-specific ploidy assessments of vegetative and reproductive organs, and vegetative propagation of putative tetraploids, followed by ploidy analysis of diverse plant tissues. Such approaches would allow rigorous evaluation of whole-plant ploidy stability and help determine whether undetected chimerism or meiotic instability contribute to the limited transmission of tetraploidy observed in common bean.

Although most of the analyzed C1 progeny were diploid, one tetraploid C1 individual (ON P2) was recovered from a preliminary assay, indicating that transmission of induced tetraploidy may occur, albeit at low frequency, in the material evaluated here. Nevertheless, the predominance of diploid progeny and the limited number of reproductive tetraploid lineages suggest that stable fixation of induced tetraploidy may represent a challenge for breeding applications in common bean. Because several tetraploid plants failed to produce seeds and the number of lineages available for inheritance analysis was limited, these observations should be interpreted cautiously and do not constitute a population-level estimate of tetraploidy transmission frequency.

From an applied perspective, these results indicate that colchicine-induced polyploidy is unlikely to serve as a reliable strategy for targeted improvement of stomatal or physiological traits in common bean. While the approach generates substantial phenotypic diversity, this variability is largely unpredictable and poorly heritable, limiting its direct use in breeding programs. However, polyploid induction may still be valuable as a tool to generate diversity from which rare, favorable genotypes could be selected, although this would require extensive screening and stabilization efforts.

These findings align with the broader perspective that polyploidy often represents an evolutionary dead end at the lineage level, despite occasionally enabling long-term innovation in specific cases [56]. In the context of common bean, our results suggest that induced polyploidization functions primarily as a generator of phenotypic dispersion rather than a directional improvement strategy, and that the predominance of diploid progeny limits the use of colchicine-induced polyploidization as a breeding technique in this crop species.

## 4. Materials and Methods

### 4.1. Plant Material

Seeds of *P. vulgaris* cv. ‘Zorzal’ were obtained from a local producer (Lora, Maule Region, Chile). Prior to polyploidization treatment, the seeds were rinsed thoroughly with distilled water.

For all experiments, control plants were established from seeds treated exclusively with distilled water and sown simultaneously with those subjected to colchicine exposure.

### 4.2. Colchicine Polyploidy Induction

Two experimental treatments were evaluated: overnight soaking in colchicine, and germination with colchicine solution.

For the overnight soaking, the seeds were immersed in a flask with a 0.1% (*w*/*v*) colchicine (Merck, Darmstadt, Germany) solution, covered with aluminum foil and incubated overnight under constant agitation. The colchicine solution was freshly prepared for each application. Following treatment, the seeds were transferred to a sterile substrate composed of peat moss and vermiculite (3:1, *v*/*v*) and irrigated with a Hoagland nutrient solution. Six independent replicates were conducted, each consisting of 30 seeds.

For the germination with the colchicine treatment, the seeds were placed between two layers of paper towel and covered with two additional layers of the same material. The assembly was then saturated with 50 mL of 0.1% (*w*/*v*) colchicine solution and maintained in plastic containers wrapped with aluminum foil to prevent light exposure. Three exposure durations were evaluated: 24 h, 48 h, and 1 week. For the 24 h and 48 h treatments, five independent replicates were conducted (30 seeds per replicate), while six independent replicates (30 seeds each) were performed for the 1-week treatment. The colchicine solution was reapplied every two days to ensure consistent exposure.

For both treatments, after germination, the seeds were transferred to the sterilized substrate (peat moss:vermiculite (3:1)) in a shed house and fertigated with the Hoagland solution. Germination was defined as the emergence of a radicle of approximately one centimeter in length.

For subsequent analyses, putative tetraploid plants were labeled by treatment group, followed by an identification number specific to the individual plant within a replicate. The following abbreviations were applied: ON (overnight soaking), I24 (imbibition for 24 h), I48 (imbibition for 48 h), and I1w (imbibition for 1 week).

The generation directly exposed to colchicine is hereafter referred to as C0, and the progeny derived from these plants are designated as C1.

### 4.3. Ploidy Analysis by DNA Flow Cytometry

Fresh leaves were collected from the bottom, middle, and top positions of both the control (untreated) and colchicine-treated plants. A 0.64 cm^2^ disc was taken from each one of the leaves. The same amount of leaf tissue from untreated plants was used as a standard for diploidy. When polyploidy was detected in the leaf samples, an individual leaf analysis was performed for each one of the three leaves to exclude sectorial chimerism within the same plant.

Leaf tissue was processed using the razor blade chopping method from Galbraight et al. [57] and the protocol from Arumuganathan and Earle [58], with the modifications from Jarpa-Tauler et al. [59].

The samples were incubated on ice for 15 min in darkness and nuclear DNA was measured using a CyFlow Ploidy Analyser flow cytometer (Sysmex Partec GmbH, Germany), with a 532 nm green laser and red-sensitive photomultiplier tube with a 590 long-pass filter. More than 10,000 events were measured per sample.

### 4.4. Stomatal Traits

The stomatal pore area (SPA), SD, and SPI were measured from the abaxial surface of three different leaves from the lower, middle, and upper parts of plants that had been confirmed to be tetraploid by DNA flow cytometry and their respective controls on the pre-anthesis stage. Fresh epidermal peelings were incubated in a stomatal opening solution, as previously described [60], and photographed with a bright field microscope coupled to a digital camera. Images were processed using FIJI software 1.54f [61].

Stomatal length (SL) and stomatal width (SW) were measured on three independent leaves per plant, with at least 10 stomata quantified per leaf. Leaf-level values were averaged to obtain one biological value per plant.

The stomatal pore area was measured based on SL and SW, approximating the calculation of the area of an ellipse, according to Mano et al. [62]:

$$SPA=\;\pi \left(\frac{SL}{2}\right)\left(\frac{SW}{2}\right)$$

Stomatal density was calculated as the mean number of stomata per mm^2^ from three different fields per leaf. The stomatal pore area index (SPI) was calculated using SPA and SD. SPA values were first converted from μm^2^ to mm^2^ to match the units of SD, which was expressed as stomata mm^−2^. The SPI was then calculated as:

SPI=SPA×SD

where the SPA is expressed in mm^2^ and the SD in stomata mm^−2^. Thus, the SPI represents a dimensionless index estimating the proportion of the leaf area theoretically occupied by stomatal pores. For graphical presentation, the SPI values were multiplied by 100. 

### 4.5. Physiological Parameters

The net photosynthesis rate (A), g_s_, E, and C_i_ were measured from intact leaves of similar sizes and ages from the mid-third of the plant without detaching them. The intrinsic water-use efficiency (WUE_i_) was calculated as the ratio of Pn and g_s_. Measurements were conducted using a portable photosynthesis system (LI-6400 XT, LI-COR Bioscience, Inc., Lincoln, NE, USA), under controlled conditions: light (800 µmol photons m^−2^ s^−1^), temperature (25 °C), and CO_2_ (400 ppm). Data collection occurred between 9 and 11 a.m., during the pre-anthesis stage.

### 4.6. Chlorophyll and Carotenoid Profiles

Chlorophylls and carotenoids were extracted from leaves using pure acetone (100% *v*/*v*, HPLC grade), following the method described by García-Plazaola and Becerril [63]. Pigment analysis was conducted by high-performance liquid chromatography (HPLC) on an Agilent Technologies 1200 series system with a Waters Spherisorb C-18 column (5.0 µm ODS1, 4.6 × 250 mm). Standard pigments, including violaxanthin, antheraxanthin, zeaxanthin, neoxanthin, chlorophyll a, chlorophyll b, β-carotene, and lutein, were obtained from Sigma-Aldrich (Sigma Chemical Co., St. Louis, MO, USA).

### 4.7. Lipid Peroxidation Assay

Malondialdehyde (MDA) was measured using the thiobarbituric acid reactive substances (TBARS) assay for plant tissue, as described by Du and Bramlage [64]. Three leaves per treatment were removed using the same criteria as in previous measurements and immediately frozen in liquid nitrogen and stored at −80 °C until analysis. The absorbance was measured at 532, 600, and 440 nm with the objective of correcting the interference generated by TBARS–sugar complexes, according to the following formula:

$$MDA=\left[\left(A_{532}-A_{600\;}\right)-\left(A_{440}-A_{600}\right)\times 0.0571\right]\times 6.37$$

The results were expressed as MDA equivalents (nmol MDA g^−1^ fresh weight (FW)) [65]. 

### 4.8. Antioxidant Determination

Antioxidant activity was determined using the 2.2-diphenyl-1-picrylhydrazyl (DPPH) free radical scavenging assay, according to the method of Chinicci et al. [66]. Samples were ground and soaked in 1 mL of 80:20 (*v*/*v*) methanol/water. Absorbance was measured at 515 nm in a spectrophotometer (UNICOR 2800 UV/VIS, Spain) using Trolox as standard. The values are expressed in μg Trolox equivalents g^−1^ FW.

### 4.9. Seed Production and Ploidy Inheritance Determination

Seed set in the polyploid plants was assessed by quantifying the total number of seeds produced per individual. Where seed availability permitted, at least 20 C1 plants per seed-producing C0 parent were evaluated by flow cytometry to assess transmission of the induced ploidy level. This sampling was intended to detect common transmission of tetraploidy rather than to estimate rare transmission events or segregation ratios with high precision. In line I1w-198, seed production was limited to 14 seeds, of which 11 developed into C1 plants and were available for ploidy determination. The seeds were sown, and the ploidy of these plants in the vegetative stage was evaluated, as previously described.

### 4.10. Statistical Analysis

Statistical analyses were performed in R version 4.3.0 (R Core Team, Vienna, Austria) and GraphPad Prism version 8.0.0 for Windows, GraphPad Software, San Diego, CA, USA.

The polyploidy and mixoploidy induction rates were calculated for each independent assay replicate as the percentage of polyploid or mixoploid plants relative to the number of germinated seeds. Differences among colchicine treatments were evaluated by one-way analysis of variance (ANOVA), using assay replicates as the experimental unit. The coefficient of variation was calculated for the induction rate of polyploidy or mixoploidy for each colchicine treatment.

For the confirmed tetraploid C0 plants, stomatal, gas-exchange, pigment, and oxidative/antioxidant traits were first summarized at the line level from the within-plant subsamples obtained for each assay. Stomatal measurements derived from stomata and fields per leaf were averaged at the leaf level and then summarized per plant. Likewise, gas-exchange, pigment, and oxidative/antioxidant measurements were expressed as the mean ± standard deviation for each evaluated line. Because each tetraploid line corresponded to an individual colchicine-derived C0 plant, downstream comparisons among the tetraploid lines were interpreted primarily as exploratory phenotypic profiling, rather than as population-level inference.

To integrate the phenotypic dataset, a numerical matrix was assembled, including stomatal anatomical traits (SPA, SD, and SPI), gas-exchange variables (A, g_s_, E, C_i_, and WUE_i_), pigment-related traits, and oxidative/antioxidant variables (TBARS, DPPH radical scavenging, and total phenolic content). Prior to the multivariate analyses, all variables were standardized using z-scores to remove scale effects and allow direct comparison among traits with different units and ranges.

Associations among variables were explored using Pearson correlation coefficients, which were visualized as a correlation matrix. To summarize the major axes of coordinated phenotypic variation across the control and tetraploid lines, principal component analysis (PCA) was performed on the standardized dataset using the line means. This multivariate ordination is presented in Figure 4. In addition, an auxiliary PCA restricted to gas-exchange variables (A, g_s_, E, and C_i_) was performed to derive an integrated gas-exchange performance index, defined as the first principal component (PC1). The scores from this gas-exchange PCA were used to rank genotypes, and the resulting ranking is shown in Figure 6.

Overall similarity among lines was further explored by hierarchical clustering based on Euclidean distances calculated from the standardized trait matrix, using Ward’s linkage method. Multivariate analyses and graphical outputs were generated using the R packages factoextra, corrplot, cluster, and vegan.

Whenever formal hypothesis testing was applied, statistical significance was considered at *p* ≤ 0.05. However, line-level analyses derived from single colchicine-generated C0 plants were interpreted conservatively as descriptive or exploratory, and emphasis was placed on integrated multivariate structure and trait coordination, rather than on broad inferential generalization.

## 5. Conclusions

In this study, colchicine-induced polyploidy in common bean resulted in phenotypic variability across stomatal, physiological, pigment, and redox-associated traits; however, it did not produce consistent or directional improvements in the targeted traits. Although individual tetraploid lines demonstrated enhanced gas-exchange performance or more coordinated metabolic profiles, these responses were highly event-specific and frequently associated with trade-offs, particularly between carbon assimilation and water-use efficiency. Notably, the expected anatomical drivers of improved water-use behavior, such as reductions in the stomatal pore area index, were not consistently achieved.

A major limitation identified in this study was the limited transgenerational stability of the induced polyploid state. Although one tetraploid C1 individual was recovered, most evaluated progeny from the seed-producing tetraploid plants were diploid. Together, these findings indicate that colchicine-induced polyploidization in common bean cv. ‘Zorzal’ acts primarily as a generator of phenotypic variability rather than as a reliable strategy for targeted trait improvement under the conditions evaluated in this study. While this variability may provide a source of favorable phenotypes, the predominance of diploid progeny among the evaluated material, coupled with the highly genotype-dependent nature of the observed responses, may limit its immediate application in breeding programs. Furthermore, because neither meiotic behavior nor tissue-specific ploidy composition was directly evaluated, the mechanisms underlying the observed instability remain unresolved. Future efforts should focus on understanding the factors governing polyploid stability and inheritance, as well as on alternative or complementary approaches capable of producing stable and heritable modifications in traits related to drought adaptation and physiological performance.

## Figures and Tables

**Table 1:** Gas-exchange parameters for the control and tetraploid lines. The mean values ± SD are shown for A, g_s_, E, C_i_, and WUE_i_. Statistical significance versus the control is indicated by asterisks (* *p* ≤ 0.05, ** *p* ≤ 0.01).

Genotype	Pn (µmol CO_2_ m^−2^ s^−1^)	g_s_ (mol H_2_O m^−2^ s^−1^)	E (mmol H_2_O m^−2^ s^−1^)	C_i_ (µmol mol^−1^)	WUE_i_ (µmol CO_2_ mol^−1^ H_2_O)
Control	4.23 ± 0.22	0.099 ± 0.002	2.44 ± 0.04	313.25 ± 4.47	42.85 ± 2.83
ON P2	11.89 ± 0.23	0.440 ± 0.001	5.33 ± 0.01	338.27 ± 1.02 **	27.06 ± 0.57 *
ON1	1.86 ± 0.08	0.047 ± 0.001	0.88 ± 0.01	323.33 ± 2.96	39.99 ± 1.89
I24-88	8.15 ± 0.16	0.212 ± 0.001	6.01 ± 0.02	322.21 ± 1.66	38.44 ± 0.99
I24-94	8.47 ± 0.12	0.191 ± 0.001	3.19 ± 0.01	312.37 ± 1.38	44.37 ± 0.82
I24-102	12.76 ± 0.13	0.487 ± 0.002 *	2.98 ± 0.02	338.85 ± 0.70 **	26.21 ± 0.36 **
I24-137	8.72 ± 0.30	0.268 ± 0.001	5.84 ± 0.02	331.01 ± 2.02 *	32.59 ± 1.21
I24-147	8.94 ± 0.19	0.238 ± 0.003	3.80 ± 0.01	323.22 ± 1.83	37.56 ± 1.19
I24-164	11.11 ± 0.22	0.351 ± 0.001	3.49 ± 0.03	331.62 ± 1.25 *	31.62 ± 0.67 *
I24-166	6.53 ± 0.07	0.155 ± 0.003	4.55 ± 0.001	317.27 ± 1.96	42.10 ± 1.18
I48-85	13.56 ± 0.28 *	0.367 ± 0.003	2.48 ± 0.04	321.18 ± 1.58	36.93 ± 1.02
I1w-2	11.73 ± 1.17	0.545 ± 0.002 **	4.73 ± 0.03	347.46 ± 3.77 **	21.50 ± 2.10 **
I1w-197	16.51 ± 0.65 **	0.558 ± 0.001 **	6.24 ± 0.01	330.87 ± 2.24 *	29.59 ± 1.11 *
I1w-198	16.37 ± 0.14 **	0.630 ± 0.004 **	6.89 ± 0.03 *	336.50 ± 0.25 *	25.97 ± 0.10 **
I1w-199	13.02 ± 0.34 *	0.450 ± 0.001 *	5.53 ± 0.01	334.40 ± 1.28 *	28.92 ± 0.69 *
I1w-227	8.66 ± 0.03	0.337 ± 0.001	4.44 ± 0.001	342.83 ± 0.22 **	25.72 ± 0.15 **

**Table 2:** The chlorophyll and carotenoid content of the tetraploid common bean plants. Chlorophyll *a*, chlorophyll *b*, and carotenoids (β-carotene, lutein, neoxanthin, vioaxanthin, and antheraxanthin) measured in the leaves of tetraploid common bean plants. The values are expressed as µg·g^−1^FW and represent the means ± SD. Significant differences are presented with asterisks (* *p* ≤ 0.05, ** *p* ≤ 0.01, *** *p* ≤ 0.001). The Kruskal–Wallis test, followed by Dunn’s multiple comparisons test. Only comparisons versus the control are shown.

Genotype	Chlorophyll *a* µg·g^−1^FW	Chlorophyll *b* µg·g^−1^FW	β-Carotene µg·g^−1^FW	Lutein µg·g^−1^FW	Neoxanthin µg·g^−1^FW	Violaxanthin µg·g^−1^FW	Antheraxanthin µg·g^−1^FW
Control	994.2 ± 224.5	229.4 ± 36.1	68.9 ± 12.2	138.3 ± 33.3	53.0 ± 8.7	36.2 ± 2.6	28.3 ± 10.9
ON P2	1243.7 ± 28.2	347.3 ± 16.7	69.1 ± 4.2	150.6 ± 8.7	62.6 ± 1.6	103.1 ± 1.6	18.6 ± 0.8
ON1	1103.4 ± 70.0	326.7 ± 19.7	62.6 ± 4.4	174.9 ± 5.8	67.4 ± 4.8	18.2 ± 1.5	20.8 ± 1.7
I24-88	1653.0 ± 112.7 **	504.7 ± 19.6 ***	113.4 ± 11.3	239.7 ± 27.4 *	104.0 ± 10.4 *	123.3 ± 3.6 *	15.3 ± 1.0
I24-94	1263.1 ± 58.4	351.0 ± 17.0	85.5 ± 3.8	149.8 ± 3.8	67.8 ± 3.0	34.3 ± 2.2	17.3 ± 0.5
I24-102	1298.8 ± 57.0	379.4 ± 23.5	84.3 ± 3.4	178.9 ± 5.4	72.3 ± 3.2	100.6 ± 5.9	22.9 ± 1.9
I24-137	1527.5 ± 77.3	452.1 ± 11.0 **	92.5 ± 6.4	189.9 ± 7.7	87.5 ± 6.7	127.4 ± 5.9 *	24.9 ± 3.0
I24-147	1182.1 ± 34.1	326.8 ± 15.0	75.7 ± 3.9	146.5 ± 3.0	66.5 ± 2.3	88.8 ± 5.1	16.7 ± 1.3
I24-164	1408.0 ± 118.5	435.3 ± 46.4 *	99.9 ± 7.0	210.4 ± 17.0	92.1 ± 7.1	111.8 ± 9.3	25.1 ± 1.7
I24-166	1125.5 ± 42.3	318.2 ± 16.5	76.2 ± 2.1	137.8 ± 1.4	62.4 ± 3.1	55.6 ± 6.6	21.3 ± 2.7
I48-85	1475.2 ± 29.7	422.9 ± 15.0 *	96.1 ± 1.0	189.9 ± 1.4	80.3 ± 1.2	87.2 ± 4.0	12.3 ± 1.1
I1w-2	1271.5 ± 98.9	348.9 ± 30.8	86.6 ± 6.1	170.5 ± 9.2	74.9 ± 3.1	88.5 ± 1.9	40.5 ± 0.8
I1w-197	1624.1 ± 22.1 **	438.5 ± 4.3 **	105.3 ± 2.2	200.5 ± 10.3	89.2 ± 2.2	101.9 ± 3.9	20.0 ± 1.5
I1w-198	1472.8 ± 43.6	408.0 ± 9.1	96.1 ± 3.1	187.1 ± 2.4	81.7 ± 2.2	129.7 ± 8.9 *	21.5 ± 0.5
I1w-199	1432.3 ± 37.6	394.4 ± 10.2	95.0 ± 3.6	163.2 ± 4.9	78.9 ± 2.5	92.4 ± 4.2	12.2 ± 1.7
I1w-227	1461.2 ± 21.1	415.9 ± 9.4	100.2 ± 3.5	188.2 ± 5.7	83.1 ± 4.6	96.9 ± 7.8	13.1 ± 1.5

**Table 3:** Oxidative damage and antioxidant capacity. Significant differences are presented with asterisks (* *p* ≤ 0.05, ** *p* ≤ 0.01).

Genotype	TBARS (nmol MDA g^−1^ FW)	DPPH (µmol Trolox eq g^−1^ FW)	Total Phenols (µg Chlorogenic Acid eq g^−1^ FW)
Control	16.93 ± 0.60	722.0 ± 8.78	679.0 ± 18.36
ON P2	23.57 ± 1.10	956.5 ± 5.12	6454.3 ± 167.5 **
ON1	55.47 ± 1.48	510.0 ± 8.85 *	514.0 ± 15.0
I24-88	19.23 ± 0.50	920.7 ± 82.47	845.7 ± 61.44
I24-94	18.03 ± 0.90	537.7 ± 5.48	642.0 ± 7.21
I24-102	24.83 ± 0.57	555.0 ± 36.79	742.3 ± 54.04
I24-137	28.97 ± 0.59	974.5 ± 24.05	677.7 ± 8.14
I24-147	19.30 ± 1.56	569.4 ± 7.30	1060.3 ± 5.86
I24-164	18.00 ± 0.89	505.4 ± 7.36 *	736.0 ± 46.78
I24-166	27.83 ± 0.67	533.9 ± 6.79	770.0 ± 10.15
I48-85	27.83 ± 2.40	1106.2 ± 33.34	324.0 ± 16.70
I1w-2	23.07 ± 0.87	965.6 ± 91.38	297.3 ± 13.01
I1w-194	26.13 ± 0.25	933.9 ± 48.71	780.7 ± 78.53
I1w-197	26.13 ± 1.15	905.2 ± 5.82	910.3 ± 2.08
I1w-198	21.17 ± 0.40	597.8 ± 9.11	1081.3 ± 14.47 *
I1w-199	19.10 ± 0.17	660.3 ± 8.09	444.0 ± 52.92
I1w-227	21.63 ± 0.47	640.3 ± 17.56	629.0 ± 7.55

**Table 4:** Poliploidy analysis of the C1 plants. The number of seeds produced by the colchicine-treated plants (C0), the total number of evaluated plants developed from the C0 seed production, and the detected ploidy of those seeds.

C0 Plants	Produced Seeds	Evaluated C1 Plants	C1 Ploidy
ON1	0	0	-
I24-88	0	0	-
I24-94	34	23	Diploid
I24-102	0	0	-
I24-137	0	0	-
I24-147	79	27	Diploid
I24-164	22	22	Diploid
I24-166	68	23	Diploid
I48-85	90	27	Diploid
I1w-2	0	0	-
I1w-194	98	23	Diploid
I1w-197	95	27	Diploid
I1w-198	14	11	Diploid
I1w-199	59	27	Diploid
I1w-227	102	27	Diploid

## Data Availability

The original contributions presented in this study are included in the article. Further inquiries can be directed to the corresponding author.

## Abbreviations

The following abbreviations are used in this manuscript:

A	Net photosynthesis rate
C_i_	Substomatal carbon concentration
DPPH	2.2-diphenyl-1-picrylhydrazyl
E	Transpiration
g_s_	Stomatal conductance
MDA	Malondialdehyde
PCA	Principal component analysis
SD	Stomatal density
SL	Stomatal length
SPA	Stomatal pore area
SPI	Stomatal pore area index
SW	Stomatal width
TBARSs	Thiobarbituric acid reactive substances
WUE_i_	Intrinsic water-use efficiency
